# Proinflammatory cytokines in a mouse model of central retinal artery occlusion

**Published:** 2009-05-01

**Authors:** Michal Kramer, S. Dadon, M. Hasanreisoglu, Y. Monselise, B. R. Avraham, A. Feldman, I. Eldar, D. Weinberger, N. Goldenberg-Cohen

**Affiliations:** 1 Department of Ophthalmology, Rabin Medical Center, Petah Tiqwa, Israel; 2 Krieger Eye Research Laboratory, Felsenstein Medical Research Center, Petah Tiqwa, Israel; 3 Laboratory of Clinical Immunology, Rabin Medical Center, Petah Tiqwa, Israel; 4 Neurogenomic laboratory, Multiple Sclerosis Center, Sheba Medical Center, Tel-Hashomer, Israel; 5 Sackler School of Medicine, Tel Aviv University, Tel Aviv, Israel

## Abstract

**Purpose:**

To analyze cytokines in the retina and serum in an experimental model of central retinal artery occlusion (CRAO) in mice.

**Methods:**

CRAO was induced by laser activation of intravenously injected rose bengal, a photosensitive dye, in 60 C57Bl/6 mice. mRNA and protein levels of macrophage inhibitory protein-2 (MIP-2), interleukin-6 (IL-6), and tumor necrosis factor- α (TNF-α) were analyzed using real-time polymerase chain reaction, and western blot, respectively. Cytokine levels in serum were measured by ELISA. Analysis was performed at various time intervals from CRAO induction.

**Results:**

In the retina, *MIP-2* and *IL-6* mRNA expression decreased 3 h after induction of CRAO and increased thereafter, peaking at 12–24 h. By 7 days, levels were again mostly undetectable. *TNF-α* mRNA expression increased at 3 h and decreased to control levels at 7 days. At the protein level, all cytokines were present at 3 h, following similar patterns to their respective gene expression thereafter. In serum, MIP-2 and TNF-α levels peaked early, and decreased to control levels at 12 h, with a second late rise of TNF-α. IL-6 levels increased between 3 and 12 h and decreased at 24 h.

**Conclusions:**

Temporal variations in cytokines were observed following the induction of CRAO, both at the retinal mRNA expression and protein levels. These temporal changes, and the variable effects of the cytokines at the different time intervals, should be taken into account during the formulation of therapeutic strategies.

## Introduction

Acute central retinal artery occlusion (CRAO) can cause severe and irreversible visual loss. The outcome depends on the vessel occluded and the duration of the occlusion [1]. In an experimental model of CRAO in rhesus monkeys, Hayreh et al. [2] demonstrated a retinal tolerance to acute ischemic occlusion lasting up to 100 min. However, occlusion longer than 240 min caused massive irreversible retinal damage, with total optic nerve atrophy and nerve fiber layer loss [2–4]. Understanding the mechanisms underlying the temporal differences in ischemic damage can help researchers develop appropriate interventions.

The role of inflammation in the pathogenesis of spontaneous and experimentally induced ischemic events is well established [5–8]. Arterial occlusion triggers tissue ischemia and a subsequent inflammatory reaction by the production of cytokines and adhesion molecules, either locally or systemically [9,10]. An elevation in inflammatory marker levels has been reported following acute ischemic events in various organs [5,6], including the eye [11,12]. Thrombotic events have been specifically correlated with an increase in the proinflammatory cytokines, interleukin 8 (IL-8), tumor necrosis factor alpha (TNF-α), and interleukin 6 (IL-6) [12–15]. Changes in the levels of these cytokines were found within minutes to hours of the ischemic event [16,17].

In previous clinical studies, we reported changes in the levels of the proinflammatory cytokines in the aqueous humor and serum of patients with CRAO [12] and in the serum of patients with anterior ischemic optic neuropathy [11]. We assumed that temporal changes in the levels of these cytokines in the aqueous humor may reflect local changes in the ischemic retina [18].

Since clinical studies of CRAO are limited by the rarity of the event and the availability of tissue, in the present study, we examined the temporal changes in proinflammatory cytokines in an experimental model of CRAO. Researchers have described the technique of laser photoactivation of an injected dye to induce retinal artery occlusion in rabbits [19] and rats [20,21]. We modified the model of Daugeliene et al. [20], which involves the injection of rose bengal, a photosensitive dye that releases active oxygen radicals when irradiated by a green light. In a previous study, we validated the experimental model and described the clinical, angiographic, histologic, and molecular changes of CRAO in mice [22]. Owing to the similarities found to human CRAO, we were able to apply the model to the investigation of additional parameters of this ischemic condition. In the present study, we analyzed the gene expression of proinflammatory cytokines in the retina, verified at the protein level, and the levels of the same cytokines in the serum, at different time points after induction of CRAO, and then correlated the cytokine profile with previous findings in clinical and experimental ocular ischemic conditions.

## Methods

All protocols were conducted in accordance with the ARVO Statement for the Use of Animals in Ophthalmic and Vision Research. Every animal protocol was approved and monitored by the Animal Care Committee of Rabin Medical Center.

### CRAO mouse model

Adult male C57bl/6 mice (25–30 g) purchased from Harlan Laboratories (Jerusalem, Israel) were housed under a 14 h light/10 h dark cycle with standard chow and water ad libitum. CRAO was induced in 60 C57Bl/6 mice. This model was previously validated in our laboratory on the basis of clinical findings, fluorescein angiography, and histopathologic study, and further characterized by ischemic gene expression [22].

The right eye of each animal was treated, and the left eye served as an internal control for retinal levels of expression. Negative controls for serum levels consisted of naive mice and mice injected with rose bengal without laser treatment. Findings were also compared between mice with moderate or severe occlusion.

### Induction of mouse CRAO

The method of CRAO induction was described in detail in our previous report [21]. Briefly, CRAO was induced in C57Bl/6 mice by laser photoactivation of intravenously injected rose Bengal (Sigma Aldrich, St. Louis, MO) dye (0.05 ml of 2.5 mM). The laser beam was directed at the central retinal artery, at the point at which it emerges from the optic nerve head. The retina was visualized directly with the help of a custom-designed fundus corneal contact lens. YAG laser (514 nm) was used, with the following specifications: 200 μm spot size, 20 shots of 0.1 s duration, 100 mW power for moderate occlusion, and 150 mW power for severe occlusion.

### Quantification of cytokine expression in the retina

#### mRNA expression

The expression of the proinflammatory cytokines in the retina was measured at 3 h, 12 h, 24 h, and 7 days after CRAO induction. Immediately after euthanasia, using CO2 inhalation, the eyes were enucleated and the retinas were snap frozen in liquid nitrogen. Total RNA was extracted using TRIzol^TM^ reagent (Invitrogen, Life Technologies, Carlsbad, CA) and reverse-transcribed into cDNA using random hexamers (Amersham Biosciences, Little Chalfont, UK) and MMLV-reverse transcriptase (Promega, Madison, WI). cDNA was analyzed by real time RT–PCR using the Sequence Detection System (ABI Prism 7900; Applied Biosystems, Inc. Foster City, CA). The expression of *MIP-2* (the murine equivalent of human IL-8) and of *IL-6*, and *TNF-α* was measured. Mouse beta-actin (ACTB) was used as the reference gene to normalize cDNA input levels. The primer pairs are shown in Table 1. Reactions were performed in a 20 µl volume containing 4 µl cDNA, 1 µl each of forward and reverse primers, and buffer included in the master mix (SYBR^®^ Green I; Applied Biosystems, Inc.). PCR cycling conditions were as follows: initial denaturation step of 95 °C for 10 min followed by 40 cycles of 1 min denaturation at 95 °C and 1 min of annealing and extension at 60 °C. Duplicate transcriptase-based quantitative PCR (RT-QPCR) reactions (Prism 7900; Applied Biosystems) were performed for each cytokine to minimize individual tube variability, and an average was taken for each time point. Standard curves for PCR assays were obtained using cDNA extracted from tissues normally producing each cytokine in naive mice (kidney for MIP-2, heart for IL-6, and spleen for TNF-α). Results were quantified and presented as relative values of the nonischemic eye using a comparative C_t_ (2^-ΔΔCt^) method [23], where ΔΔC_t_=ΔC_t_ (sample)–ΔC_t_ (reference gene).

**Table 1:** Primer sequences

**Gene**	**Forward (5′3′)**	**Reverse (3′5′)**
*TNF-α*	TCTCAAAATTCGAGTGACAAGC	ACTCCAGCTGCTCCTCCAC
*IL-6*	GTTCTCTGGGAAATCGTGGA	TTCTGCAAGTGCATCATCGT
*MIP-2*	GCGCCCAGACAGAAGTCATAG	GGCAAACTTTTTGACCGCC

Enlisted in the table are the pairs of primers for each of the following genes (*TNF-α, IL-6, MIP-2*) used for RT PCR to measure levels of mRNA expression in the mouse retina.

#### Protein levels using Western-blot analysis

Protein analysis was performed from 3 to 5 retinal samples of the same mice, at each of the following time points: 3 h, 12 h, and 7 days. All equipment for protein gel electrophoresis was purchased from Biorad Laboratories (Hercules, CA). Proteins were in Trizol® (Invitrogen), following mRNA extraction, therefore an appropriate protocol was used. Due to the small quantity of extracted material which was previously analyzed for gene expression, the samples of each time point were pooled. Protein samples were prepared and separated using a 12% acryl-amide SDS–PAGE gel. Nitrocellulose membranes were prepared by soaking in wetting solution (25 mM tris/glycine [Biorad Laboratories], 20% methanol [Sigma Aldrich], 0.1% SDS [Biorad Laboratories] in DDW) for a 2 min and the proteins transferred upon high voltage of 400 mA (25V) per 2 cm of gel area for 2 h. Membranes were blocked with 10% BSA solution for 2 h at 4 °C. The goat anti-rat-IL-6 (19 kDa), -TNF-α (17 kDa), -MIP-2 (9 kDa) antibodies and recombinant proteins (as a positive control for each protein; eBioscience Inc.,) were diluted according to manufacturer’s recommendations, in 10% BSA (Sigma Aldrich) blocking solution. The solution was then added to the membranes and incubated on rocker/shaker at 4 °C, overnight. Membranes were washed and labeled with donkey anti-goat IgG-HRP conjugated (Jackson, West Grove, PA) antibody. The enhanced chemilumiscent (ECL; Pierce Biotechnology, Rockford, IL) reagent was added to the membranes. Films were developed and analyzed.

### Measurement of cytokine levels in serum

Serum cytokine levels were measured at 1 h, 3 h, 6 h, 12 h, 24 h, 7 days, and 21 days after CRAO induction. Immediately following euthanasia, peripheral blood was collected. The plasma was separated by centrifugation and stored at −70 °C. Levels of IL-6, TNF-α, and MIP-2 were measured simultaneously using an ELISA kit (R&D, Minneapolis, MN).

### Statistical analysis

Cytokine expression in the retina was evaluated by using the student's *t*-test, to compare the moderate and severe occlusion groups. Significance was set at p<0.05. Mann–Whitney nonparametric tests were used to compare findings between different time points. Student's *t*-test was used to compare serum cytokine levels at different time points and with control levels.

## Results

### Gene expression of cytokines in the retina

Relative quantitative analysis of cytokine expression by real time RT–PCR revealed similar trends in the mice that underwent high-intensity (150 mW power) and moderate-intensity (100 mW power) laser photoactivation. There was no statistically significant difference between them for any of the cytokines at any time point. Therefore, for the remainder of the study, these two groups were analyzed jointly.

**Figure 1:**
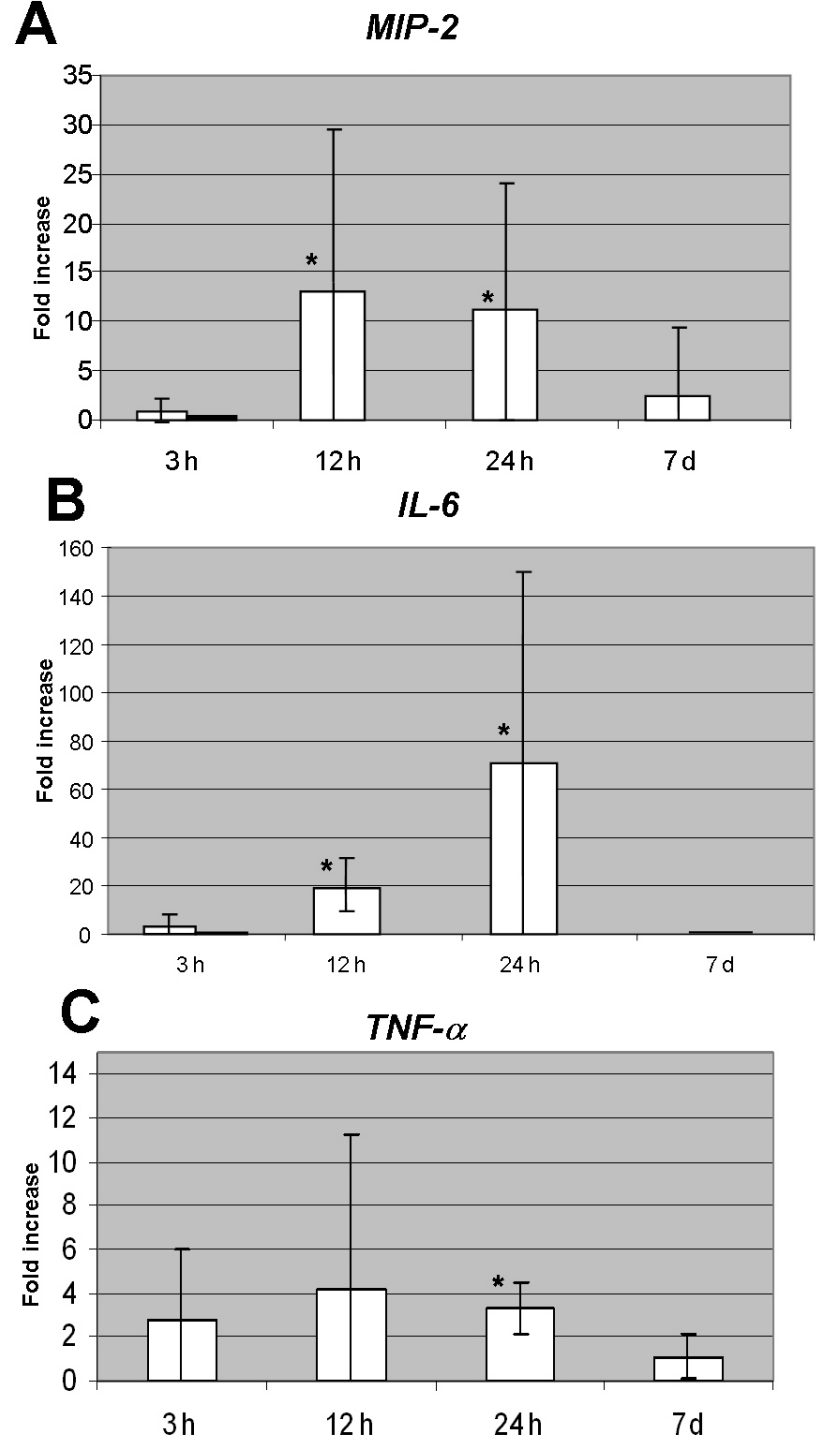
Relative mRNA expression of proinflammatory cytokines in ischemic eyes compared to control eyes at variable time intervals from CRAO induction. **A:** Elevated levels of *MIP-2* at 12 h and 24 h are statistically significantly higher than the levels at 3 h and 7 days (p<0.01). Light bar in the 3 h column represents total mean value, and the dark bar represents the trend of reduced expression. The bars at 12 h, 24 h, and 7 days display the mean values which represent the trends as well. **B:** Levels of *IL-6* peaked at 12 h and 24 h. The differences between levels at 3 h and 12 h, as well as between 3 h and 24 h were statistically significant (p<0.01, 0.02, respectively). Levels at 7 days were statistically significantly lower than levels at 24 h (p<0.01). Light bar in the 3 h column represents total mean value, and the dark bar represents the trend of reduced expression. The bars at 12 h, 24 h, and 7 days display the mean values which represent the trends as well. **C:** Level of expression of *TNF-α* at 24 h was statistically significantly different from expression at 7 days (p=0.033). The asterisk indicates statistical significance. Error bars represent SD.

Gene expression of cytokines in the ischemic eyes showed wide variability, as outlined in Table 2. Absolute expression of cytokines in the left control eyes was low to undetectable and remained so at all time points. The expression in the ischemic eyes relative to the levels in the control (untreated) eyes is shown in Figure 1.

**Table 2:** mRNA expression of cytokines in the retina: *MIP-2, IL-6, TNF-α.*

***MIP-2***			
**3 h**	**12 h**	**24 h**	**7 days**
**n=7**	**n=10**	**n=4**	**n=7**
0.65	2.3	3.93	0
−3.93	4.24	29.04	0.36
0.44	−0.3	2.11	0.03
0.08	35.3	14.12	0.39
−1.58	5.54		0.2
0.28	11		−20.4
0.21	20.25		0.16
0.49	11.43		0.6
0.34	11.43		−1.95
	3.32		
	49.69		
***IL-6***			
**3 h**	**12 h**	**24 h**	**7 days**
**n=6**	**n=8**	**n=3**	**n=7**
0.68	−1.15	56.1	0
0.6	21.86	182.91	0
−16.5	17.21	47.83	0
0.41	10.2	−1.36	0
−3.14	38.85		0.4
−3.36	25.36		0.09
0.315	21.18		0.77
0.6	30.06		
0.47	16.22		
***TNF-α***			
**3 h**	**12 h**	**24 h**	**7 days**
**n=5**	**n=7**	**n=4**	**n=6**
−0.008	−0.59	2.52	0.25
5.52	1.55	4.87	1.87
1.56	−0.63	2.24	0.37
1.01	13.74	3.49	0.15
7.97	2.11		2.58
7.62	1.25		1.42
−0.11	1.54		
−0.43	1.94		
−0.54	−0.83		
	−0.34		
	22		

Relative expression of mRNA of cytokines in the retina of the ischemic eyes, as compared to the left untreated control eye (*MIP-2, IL-6, TNF-α*), at different time intervals from CRAO induction. The marked numbers represent extreme points which did not follow the trend determined by the majority of samples at each time point. The number of samples at each time point refers to the samples which comprise the majority determining the trend.

Mean levels were calculated for all samples at each time point in the ischemic eyes. However, our analysis of the data showed that the trends in cytokine expression identified by the change in the majority of samples at each time point were not always reflected by the mean values. These trends are also presented and discussed.

**Figure 2:**
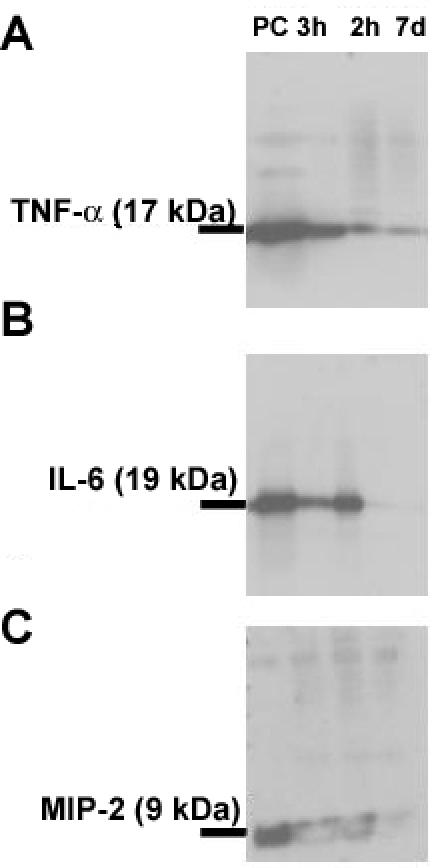
Western blot analysis of cytokines in retinal samples at different time points. Western blot analysis of the cytokines in pooled retinal samples, using goat anti–rat antibodies. Recombinant proteins were used for each cytokine as positive control. The proteins were separated using SDS–PAGE. Labeling was performed using donkey anti goat IgG HRP conjugated antibody. Films were developed using ECL reagent. **A:** Western blot analysis demonstrated the presence of TNF-α at all time points measured with high intensity bands at both 3 h and 12 h. A slight decrease occurred at 7 days. **B:** Western blot analysis of IL-6 demonstrated the presence of the cytokine in the retina at 3 h. Band intensity increased at 12 h, and was hardly visible at 7 days. **C:** Western blot analysis of MIP-2 demonstrated the presence of the cytokine in all studied samples. MIP-2 was evident at 3 h, with maximal band intensity at 12 h, and hardly visible at 7 days. PC represents positive control.

The expression of the cytokines was further verified in the retinal tissue by western blot analysis. Results are detailed for each cytokine, and presented in Figure 2.

#### MIP-2

At 3 h after CRAO induction, mean *MIP-2* mRNA expression in the ischemic eyes was 0.9±1.2 fold of the expression in the control (untreated) eyes. Detailed analysis revealed that in 7 of the 9 samples, levels were actually low, with a mean level of 0.35±0.2 fold of control level (range 0.08–0.65). At 12 h, mean levels peaked to 14±15 fold of controls (p<0.01, compared to the expression in the ischemic eyes at 3 h). Elevated expression was found in 10 of 11 samples to 2–50 fold of controls. Expression was still high at 24 h in all 4 samples examined at that time point (range 4–29 fold of controls, mean 12±12 fold; p<0.01, compared to the expression at 3 h). At 7 days, mean *MIP-2* mRNA expression significantly decreased to 2.7±6.7 fold of controls (p=0.02, compared to the expression at 24 h). In 7 of 9 samples, levels were below control levels (mean reduced levels 0.3±0.2, p<0.01; Figure 1A).

Western blot analysis demonstrated cytokine presence in all studied samples. MIP-2 was evident at 3 h, with maximal band intensity at 12 h, and hardly visible at 7 days (Figure 2)

#### IL-6

At 3 h after CRAO induction, mean expression of *IL-6* mRNA was 2.9±5.24 fold of the control level. On detailed analysis of the data, we found that in 6 of 9 samples, the relative expression of *IL-6* was actually lower (mean 0.5±0.1 fold of control levels). *IL-6* mRNA mean expression at 12 h increased to 20±11 fold of controls (p<0.01). Elevation was noted in 8 of 9 samples to 10–39-fold of controls values. Mean levels peaked at 24 h to 72±78 fold (p=0.02 compared to the expression at 3 h). Expression was high in 3 of 4 samples (48–183 fold of controls). At 7 days, the expression of *IL-6* mRNA was undetectable in most samples (p<0.01, compared to the expression at 24 h; Figure 1B).

Western blot analysis demonstrated that IL-6 was present in the retina at 3 h. Band intensity increased at 12 h, and was hardly visible at 7 days. (Figure 2)

#### TNF-α

At 3 h after CRAO induction, the relative expression of *TNF-α* in the retina increased by a mean of 2.7±3.3 fold of control values. A further elevation was noted at 12 h (mean 4.2±7.0). At 24 h, there was a gradual reduction (mean 3.3±1.2 fold), which reached near-control levels at 7 days (mean 1.1±1.0). The difference in expression at 24 h and 7 days was statistically significant (p=0.033; Figure 1C).

Western blot analysis demonstrated the presence of TNF-α in the retina at all time points measured, with high intensity at both 3 h and 12 h. A slight decrease, however, still present, at 7 days (Figure 2)

### Serum levels of proinflammatory cytokines

**Figure 3:**
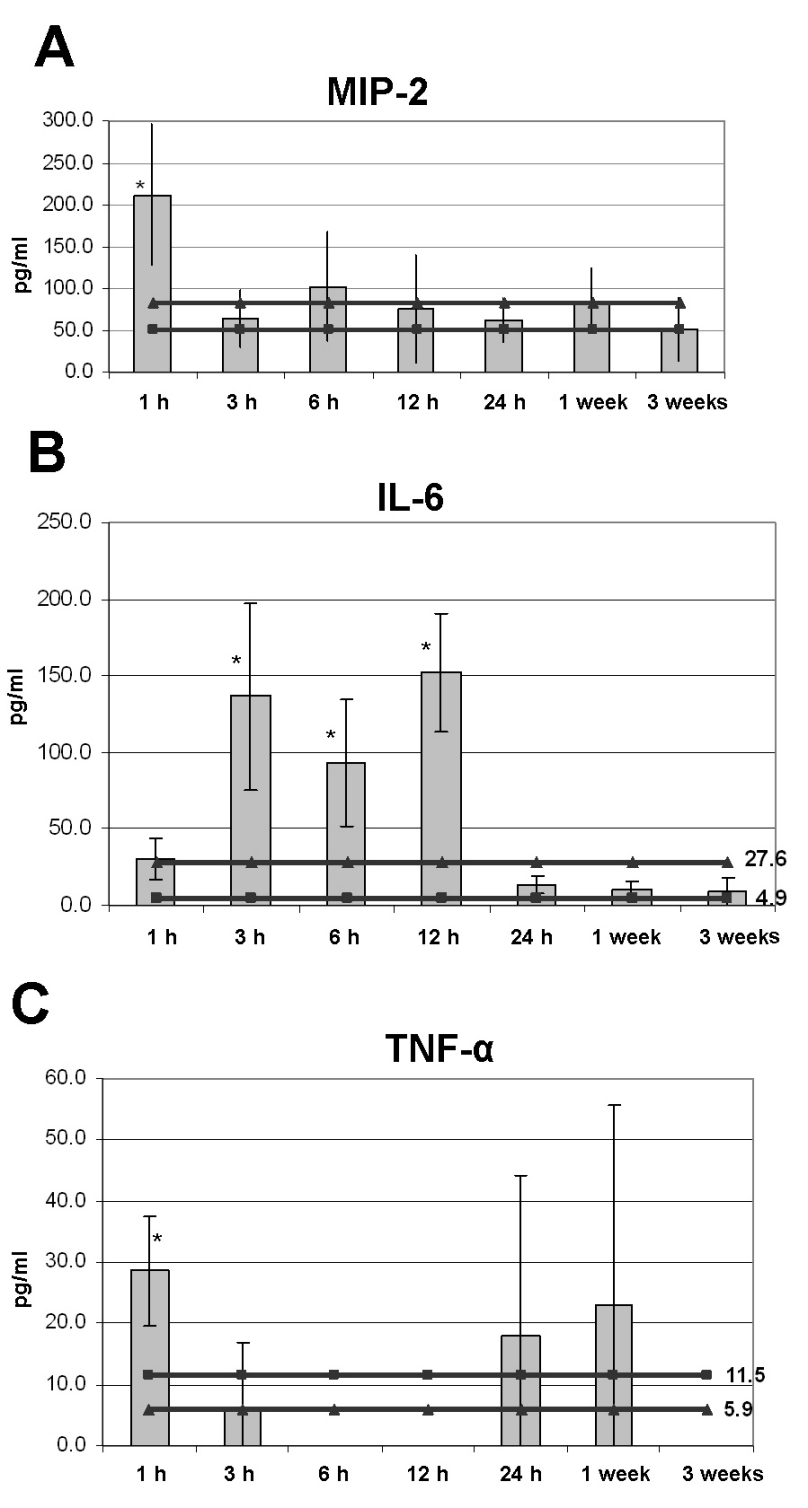
Serum levels of proinflammatory cytokines are expressed in pg/ml, relative to time interval from CRAO induction. The square-interspersed line indicates the control level in naìve mice. The triangle-interspersed line indicates the control level in rose-bengal-injected mice. Error bars represent SD. The asterisk indicates statistical significance. The specific values are detailed for each cytokine separately. **A:** MIP-2 Levels were elevated at 1 h. The level at 1 h was statistically significantly higher than levels thereafter (p=0.03). **B:** IL-6 levels increased at 3 h and remained elevated at 6 h and 12 h. The elevated levels were statistically significantly higher than levels of both control groups (p=0.02, 0.03 and 0.003 for 3 h, 6 h, and 12 h, respectively). **C:** TNF-α showed an early peak at 1h and late elevation at 1week and 3 weeks. Statistical significance was found only between the early peak and the control levels of rose bengal injected mice.

Serum levels were measured only in the mice treated with the high-intensity laser. Our previous experience in ischemic conditions revealed large variability in cytokines' blood levels. Thus, more conclusive results were expected in the more intense ischemic condition [11,12]. Three to 4 mice were used for each time point. Mean values of the temporal changes are presented in Figure 3.

#### MIP-2

Already at 1 h after CRAO induction, the mean serum level of MIP-2 was significantly elevated (212.2±83.3 pg/ml) compared to values in naive mice (50.7±31.7 pg/ml) and mice treated with rose bengal only (83.0±35.2 pg/ml; p=0.03). By 3 h, levels returned to control values, and then remained low throughout the rest of the follow-up period, up to 3 weeks after induction (Figure 3A).

#### IL-6

Serum IL-6 levels increased later than MIP-2 levels, at 3 h after CRAO induction, and they remained elevated for up to 12 h after the ischemic event. There was a statistically significant difference in IL-6 levels between the study mice and the two control groups at 3 h (136.5±61.3 pg/ml versus 4.9±3.7 pg/ml (naìve mice) and 27.6±20.8 pg/ml (rose bengal injected mice) ; p=0.02). A sharp decline in serum levels was found at 24 h, and levels remained low for the next 3 weeks (Figure 3B).

#### TNF-α

TNF-α showed a biphasic pattern in serum: Mean levels were elevated already 1 h after CRAO induction (28.54±9.1 pg/ml) compared to values in naïve mice (11.53±20.0 pg/ml; p=NS) and mice treated with rose bengal only (5.9±8.0 pg/ml; p=0.03). Additional peaks were measured at 24 h (17.9±26.4 pg/ml) and at 1 week (23.1±32.6 pg/ml; Figure 3C). The differences between TNF-α levels at 1 h, 6 h, 12 h, and 3 weeks were statistically significant (p=0.03).

## Discussion

This study demonstrates the temporal changes that take place in the levels of different proinflammatory cytokines after CRAO induction and their interrelationships, locally in the retina and systemically in the serum. Most of the changes in serum levels occurred within the first 24 h after the ischemic event (Figure 3): MIP-2 and TNF-α levels peaked early and dropped shortly thereafter, with a second late elevation of TNF-α; IL-6 rose when MIP-2 and TNF-α declined and remained high for up to 12 h. These findings are in line with previous reports of an immediate elevation in serum cytokine levels after ischemic reperfusion insult. Studies of acute myocardial ischemia reported an increase in systemic levels of IL-8, IL-6, and TNF-α [14,17,24], peaking (IL-8 and IL-6) 3 h to 6 h later [6,7]. The changes correlated with the amount of damage and disease outcome, and the systemic elevation in TNF-α served as an independent determinant of reperfusion injury after acute myocardial infarction [25]. Correlations between severity of ischemic damage and cytokines levels was also found for IL-8 and TNF-α in patients with acute stroke [26] and for IL-6 and IL-8 in asphyxiated neonates [27].

In the retina, patterns of gene expression at the mRNA levels are expressed by mean values. However, because of the presence of a few extreme values, the means may not reflect real trends of expression. Therefore, we believe that is more appropriate to determine trends using the majority of samples that show a change in a certain direction. In the present study, mRNA levels of *MIP-2* and *IL-6* were low at 3 h, while *TNF-α* levels increased. All cytokines increased at 12–24 h, and declined thereafter (Figure 1). At the protein level, all cytokines were present in the retina at 3 h, and later followed the expression pattern of their respective mRNA. Only a few previous studies measured cytokine levels in the retina in various models of eye diseases. One found that TNF-α was the most prominent cytokine after transient ischemia in the rat retina, showing an upregulation of gene expression within 0.5–48 h after the insult, and a peak at 12 h [28]. Another study localized upregulation of various chemotactic cytokines first to the retinal vasculature, and later to the inner retinal layers [29].

In an earlier study of patients with CRAO by our group [12], there was an increase in serum levels of IL-8, IL-6, and TNF-α within the first 3 h after the ischemic event, followed by a significant decline, with no temporal variance among the different cytokines. At the onset of CRAO, samples taken from the aqueous humor showed an early elevation in IL-8 and TNF-α levels followed by a sharp decline, whereas IL-6 levels increased only after 24 h. Serum levels showed the same trends as in our experimental model. Locally, TNF-α followed the same trends in the mouse retina and in patients. IL-6 and MIP-2 were present in the mouse retina early, while their mRNA was upregulated only 12 h later. Albadawi et al. [30] showed that only prolonged ischemia (6 h) followed by reperfusion could increase the steady-state levels of *IL-8* mRNA, and that during ischemia, stored IL-8 may be released nonspecifically while de novo synthesis takes over. Thus, it is possible that in patients with CRAO, the systemic elevation of IL-8 levels, as well as their early local increase, may be due initially to the release of stored intracellular IL-8, and only later to de novo synthesis. Therefore, it may also be that in our experimental model, preexisting IL-6 and MIP-2 were secreted, and detected within the retina, while new production occurred later when mRNA was upregulated.

Proinflammatory cytokines may play a role in the preservation of the tissue or in its destruction. Following ischemia-reperfusion injury, proinflammatory mediators affect neuroprotection and neurotoxicity processes [30–32]. IL-8 (or its mouse equivalent MIP-2) activates chemotaxis and adhesion of neutrophils to the endothelial cell surface, causing a paradoxical increase in the tissue injury [26,31]. An increase in IL-8 mRNA expression and immunoreactive protein levels was also reported in vascular layers of ischemic retina in rodents [29], similar to our findings of increased retinal expression of *MIP-2* mRNA 12 h after induction of ischemia, followed by increased levels of the cytokine.

Under conditions of ischemia-reperfusion, IL-6 has a neuroprotective effect [33–35] and is considered an important endogenous inhibitor of neuronal death [36]. Clinical studies have shown that IL-6 levels peak within 12 h of ischemic stroke and stimulate other protective mediators [36]. These findings were supported by experimental studies [24]. However, IL-6 may also cause neurotoxicity [37] in the first 24 h following injury. We found that *IL-6* mRNA in the retina was down-regulated early after CRAO induction. However, IL-6 was already detected within the retinal tissue. *IL-6* mRNA levels rose only after *MIP-2* and *TNF-α* mRNA levels decreased. Other researchers reported high local expression of IL-6 following ischemia in organs such as heart [5] or brain [6], and in a rat model of ocular [28] and brain ischemia [35]. It is possible that the early presence of IL-6 in the retina may exert a neurotoxic effect, but the early downregulation of *IL-6* mRNA, as well as its delayed increase, may exert a neuroprotective effect. Since in normal conditions, IL-6 levels in the eye are very low to undetectable, the systemic elevation may be caused by a remote effect rather than direct spillover from the eye.

TNF-α, an early strong immune mediator, is produced by astrocytes, microglia, and neurons. It is expressed upon exposure to various stimuli, including ischemia and ischemia-reperfusion injury. We found a biphasic elevation of TNF-α in the serum and an increase in the retina, both at the mRNA and at the protein levels within 24 h. TNF-α was reported to be upregulated in the brain after injury, and its excessive synthesis was correlated with poor prognosis; inhibition of TNF-α expression reduced brain damage [38,39]. Researchers have suggested that TNF-α induces vascular inflammation leading to vascular endothelial dysfunction [40]. It apparently induces apoptotic cell death, and, specifically, neuronal cell death in the retina via TNF-receptor −1 [41]. In cerebral ischemia, TNF-α probably has a dual function: neurotoxicity via upregulation of inducible nitric oxide synthase, and neuroprotection in the absence of nitric oxide synthase [42].

Thus, given that proinflammatory cytokines participate in both the preservation and destruction of post-ischemic tissue, therapeutic solutions should take these diverse effects, by time and conditions, into account. Some studies suggest that anti-IL-8 therapy may interfere with the mobilization of neutrophils and inflammatory cells during reperfusion, thereby reducing ischemic damage. The inhibition of IL-8 in an experimental model of transient brain ischemic injury led to a decrease in neutrophil infiltration [43]. However, since IL-8 is also involved in the process of homing and mobilizing stem cells to the area of injury, blocking its expression could have adverse effects [44].

Anti-TNF-α agents are clinically available. Their use has been found in experimental models to reduce ischemic damage in myocardium [45,46] and lung [47]. Some of the therapeutic effect of antiplatelet agents in brain ischemia involves modulation of TNF–α [26].

The present CRAO mouse model is amenable to the measurement of in situ and serum levels of expression of proinflammatory cytokines after ischemic retinal damage. However, because cytokine measurement is difficult, and baseline levels are almost undetectable, we found a high variability in the samples analyzed. When we enlarged the study group, we were able to identify trends, but had to ignore extremes; we were unable to optimize the systems even by comparing two different intensities of injury. Nevertheless, the trends detected were similar to those previously reported in patients with CRAO. A second limitation of the study was the absence of data on changes in cytokine levels in remote organs. Recently, researchers reported the upregulation of cytokines also in uninvolved tissues—for example, a high expression of TNF-α in the brain following cardiac ischemia [48] and trauma [49]. The authors speculated that this effect was mediated by the sympathetic and parasympathetic nervous systems, and not by direct blood-borne mediators.

In conclusion, the trend in the changes in cytokine expression in the retina and serum during ischemia induced by retinal artery occlusion resemble previous findings in humans and may affect the severity of damage and, thereby, outcome. The absence of *IL-6* mRNA early after the ischemic event and its later expression may indicate a protective role. MIP-2 and TNF-α may be involved in worsening the ischemic damage. Therefore, therapeutic strategies should take the timely modulation of these cytokines into account.
